# Trait preferences and lentil varietal adoption in central Ethiopia: A multistakeholder approach

**DOI:** 10.1371/journal.pone.0319152

**Published:** 2025-04-02

**Authors:** Dina Najjar, Jemima Nomunume Baada, Daniel Amoak, Dorsaf Oueslati, Shiv Kumar

**Affiliations:** 1 International Centre for Agricultural Research in the Dry Areas, Rabat, Morocco; 2 Department of Geography, University of British Columbia, Vancouver, British Columbia, Canada; 3 Department of Geography and Environmental Management, University of Waterloo, Vancouver, British Columbia, Canada; National Bureau of Plant Genetic Resources, INDIA

## Abstract

Agricultural technologies, including modern/improved crop varieties, are a critical measure for improving productivity, meeting food security needs, and bridging inequalities. This notwithstanding, adoption of some improved crop varieties in sub-Saharan Africa (SSA) tends to be low, with factors such as limited information, poor access to inputs, and risk averseness cited as reasons for low adoption. Few studies in SSA, and Ethiopia particularly, examine the influence of lentil trait preferences on adoption, and the ones that do only look at farmers’ perspectives who are often treated as a homogenous group. This is despite the importance of lentils as a subsistence and growing market crop, and the fact that diverse factors may determine adoption among farmers. To address these knowledge gaps, this study used a mixed methods approach involving multiple stakeholders (n = 808) to understand gendered patterns in lentil varietal adoption and trait preferences, using an intersectional lens. The findings revealed low adoption rates for improved varieties for women and men alike due to poor disease resistance, and insufficient attention from the breeding programs to preferred processing and consumer traits, as well as the differentiated needs of farmers. Paying attention to these trait needs serves to inform gender-intentional breeding and improve the income generation potential of lentil varieties for diverse farmer groups. As such, we recommend sex-disaggregated data collection from socially differentiated groups and market representatives in order to inform breeding priorities along with the development of multiple varieties that suit different needs.

## Introduction

Modern and/or Improved crop varieties have been identified as a critical strategy for improving farm productivity, meeting nutritional and food security needs, improving the income-earning potential of farmers, and bridging inequalities among farmers globally [1–5]. Agricultural technologies such as crop varieties are thus central to meeting these goals. In sub-Saharan Africa (SSA), findings on the adoption of improved crop varieties have been mixed. While some studies show that farmers welcome new and improved crop varieties, leading to high adoption rates, several others show that farmers in the region prefer to either grow a hybrid of local/old and improved/new crop varieties or keep producing local varieties, even with the introduction of new ones [1,3,6,7]. Several reasons have been advanced for this reluctance to grow improved crop varieties, including lack of information, poor access to inputs especially quality seeds, limited profitability of new varieties, and the risk averseness of farmers [3,7].

Some researchers reported that factors such as age, socioeconomic status (e.g., farm size, access to inputs), household (HH) type (e.g., male-headed, female-headed), and broader agricultural conditions (e.g., policies, market access) all influence the adoption of modern crop varieties [3,8–10]. For instance, Fisher and Kandiwa [2] in their study on modern maize found that women farmers had comparatively lower rates of adoption than their male counterparts. Similarly, Peterman et al. [11] and Ashby and Polar [12] show a persistent gender gap in the adoption of modern crop varieties alongside other new technologies, among women in SSA. To understand the different rates of adoption among farmers, it is crucial to first examine their differentiated trait preferences, and the underlying reasons for these preferences [12,13]. To illustrate, although many studies find that men are more likely to adopt new varieties compared to women, these gendered adoption rates have been found to be rooted in structural and sociocultural factors including gendered power dynamics, as well as access to resources and inputs such as labour, fertilizers, seeds, land and credit [2,14,15]. Given that many cultures in SSA are patriarchal with men often controlling productive resources; the ability of men to access and use new technologies may therefore be better than that of women.

Apart from the resource-differentiated nature of adoption, gender roles and responsibilities both on the farm and at home have also been shown to influence trait preferences and adoption. Trait preferences for crop varieties may differ for women and men based on the need to perform culturally ascribed responsibilities (as caregivers and breadwinners respectively), and the roles that accompany these[12,13,16,17]. Thus, to meet their responsibilities as breadwinners, men may be expected to engage in economic tasks such as market-oriented farming to raise income. A male farmer may therefore prefer market traits such as high yield, grain shape and size, and storability in a new variety. On the other hand, to satisfy caregiving responsibilities, women may be expected to perform domestic chores such as cooking, cleaning, and childcare, as well as provide on-farm (often unpaid) family labour. Consequently, women farmers may prefer traits such as good taste and energy efficiency (less cooking time) to facilitate their caregiving roles. They may also prefer traits such as less weeding and easy harvesting to make their on-farm work less cumbersome and reduce time spent on the farm [2,18,19]. Ultimately, however, as most HH heads in SSA tend to be men, they may have more decision-making power and may therefore have the final say in adoption decisions.

Nevertheless, it is important to note that, due to the increasing overlap in the roles and responsibilities of women and men farmers, traits and preferences may not always differ. For instance, Akankwasa et al. [19] found that both women and men farmers had similar quality and agronomic traits for the improved banana crop. Similarly, Ashby and Polar [12] show that women and men farmers may sometimes have similar trait preferences, but for different underlying reasons. Finally, studies show that trait preferences may not always be universal even among farmers of the same gender [14,15,19], thereby emphasizing the importance of mediating factors such as socioeconomic status, household type, and other contextual factors on trait preferences and adoption.

These contextual factors are particularly important to consider in plant breeding programs, given these programs’ aim of improving the quantity and quality of staple foods that significant proportions of the world’s population depend on for food security and economic livelihoods [8,18,20]. Also, the success of crop varietal adoption may depend on whether or not varietal traits meet the needs of targeted farmers, showcasing the need to integrate farmer preferences into breeding [16,21]. Furthermore, the role of other stakeholders’ trait preferences (e.g., agro-processors and traders) may be important to consider in breeding and adoption studies, as these may also influence farmers’ preferred traits and adoption, and the extent to which farmers may benefit from the adoption of a crop variety with specific traits [22].

Lentil is one of the oldest annual crops and a significant source of nutrition for human consumption for thousands of years. The global lentil cultivation area is approximately 6.10 million hectares, producing an annual yield of 6.33 million tons and an average yield of 1,038 kg per hectare [23]. In Ethiopia, lentil is an important food crop, widely consumed in the country and integral to food security. Recent estimates suggest that over 200,000 tons of lentils are produced each year, representing about 7.5% of the total area of cultivated [24]. With an increasing market value both globally and within Ethiopia, many men and women farmers are taking up lentil production. Emerging evidence, however, indicates that structural and gender-specific constraints significantly influence the adoption of lentil varieties and associated cultivation practices [25]. Yet, despite the importance of considering multistakeholders’ perspectives, few studies have examined lentil trait preferences and varietal adoption, and even fewer explore how farmer preferences and adoption influence the priorities/goals of breeding programs. Also, the influence of other axes of social difference – e.g., socioeconomic status and household type – on varietal adoption remains under-studied.

To address this void in the literature, this paper examines the gendered patterns and trait preferences in the adoption of lentil varieties among farmers in central Ethiopia. Specifically, it looks at trait preferences for farmers, who may also be consumers, as well as those of traders and agro-processors, to understand how these stakeholder preferences influence lentil adoption. Importantly, the study takes an intersectional approach by exploring gender, HH type and other social characteristics, to know how these affect trait preferences and adoption. An intersectional lens is necessary, as most gender analyses of trait preferences often do not examine within-group differences. The study focuses on the lentil crop, as no study has examined how traits influence lentil varietal adoption in Ethiopia.

### Theoretical perspective—intersectionality framework

This study is hinged on the intersectionality theoretical perspective. The theory, which was developed by Kimberlé Crenshaw in the late 1980s, emphasizes how standard models of discrimination—usually centred on a single marker of identity—fail to adequately represent the nuanced experiences of people who are situated within several marginalized identifies [26]. Athough intersectionality originated through critical race and feminist literature, it has become increasingly important in understanding everyday agrarian challenges, including crop breeding. According to De Dilva [27] and Quisumbing et al. [28], intersectionality as a theoretical lens interrogates how different social identities—like gender, race, class, marital status, and household type and ethnicity—intersect and operate together to produce overlapping systems of disadvantage or discrimination. It emphasizes that men and women may be marginalized to different degrees depending on how other variables interact with gender, which can either exacerbate or balance their marginalization. To this end, the foundational ideas of intersectionality are that a person is made up of several interrelated systems and identities and that these interwoven social identities have the power to influence a person’s chances and experiences [29]. It also assesses how different social identities interact to preserve and perpetuate power dynamics and structural inequities. This lens highlights the importance of context-specificity, thereby taking into account the historical, cultural, and socioeconomic environment since these elements can have a substantial impact on the effects of intersecting identities [27,30].

In the current literature, studies demonstrate how intersectionality can enhance understanding of environmental change, agrarian food systems and governance by highlighting how the combined influence of several social features results in varying access to resources among genders and even within the same gender [27,31–33]. For example, Bacud et al.’s [15] research on seed traits and varietal preferences argued that compounding degrees of marginalization, rather than gender alone drive trait preferences, with men and women having similar preferences when a combination of other markers such as higher levels of poverty are factored in. The study further asserts that there are marked differences in the traits that women with varying access to land, labour capital, authority, cast and other social markers prefer, emphasizing the importance of intersectionality in assessing within-gender differences. The intersectionality lens underscores how looking beyond gender, by considering other markers of difference, such as marital status, class and household structure (polygamy vs monogamy) as well as household headship can inform not only vulnerability to hazards but also the differentiated access to resources, including the adoption of improved agricultural innovations [4,15,34]. Unfortunately, the current literature often employs the sex of the household head as a proxy for gender, thereby homogenizing social conditions and preferences within the household, leading to the silencing of women’s choices [12,35].

The use of intersectionality is therefore most appropriate for this study as it provides a lens for examining the intricate interactions between many social variables that affect farmers in Central Ethiopia’s choice of lentil traits and varietal adoption. Conventional agriculture research frequently takes a one-dimensional approach, emphasizing just agronomic or economic variables and men-women binaries. These approaches, however, may miss important social factors that influence farmers’ choices and actions. By employing the intersectionality lens this research attempts to go beyond simplistic explanations and give a more comprehensive insight into how many, overlapping identities impact varietal trait preferences. We close these disparities by uniquely exploring how a number of non-gender-specific factors, including power dynamics, household headship, marriage status, and their intersections, lead to varietal trait preferences. The paper argues that by employing the intersectionality lens, distinct demands and challenges encountered by various farmer groups may be identified, leading to the development of more inclusive and focused agricultural policies and interventions that meet the specific needs of diverse farmers.

### Study context and methods

This study was conducted among lentil farmers, breeders, agro-processors, and traders in Ethiopia. Agriculture forms the mainstay of the Ethiopian economy, contributing 80 and 40 percent to exports and gross domestic product (GDP), respectively [36]. It also employs most of the country’s population (75%), highlighting the importance of agricultural activities in the country. The study was undertaken in North Shoa-Zone (Amhara region) and East Shoa Zone (Oromia region). The North Shoa Zone had a population of around 1,431,305, while the East Shoa Zone had about 1,356,342 residents, making them the two most populous regions in the country and together make up 60 percent of the Ethiopian population, according to the last official census [37]. Like the rest of the country, agriculture is the main economic activity in these zones, featuring both crop production and livestock rearing. Key crops include cereals like teff, wheat, barley, and maize, pulses such as beans, lentils, and chickpeas, as well as oilseeds like niger seed [36]. Additionally, vegetables and fruits are cultivated. Livestock rearing includes cattle, sheep, goats, and poultry. The agricultural practices are largely traditional, relying on manual labor and animal traction, though efforts are underway to modernize agriculture through improved seeds, fertilizers, and farming techniques. During the lentil growing season, the average rainfall in North Shoa is around 400 mm with temperatures averaging 20°C, while in East Shoa, the average rainfall is about 450 mm with temperatures averaging 22°C [38]. This diverse agricultural landscape supports the livelihoods of many smallholder farmers in the region.

Both regions share similar socioeconomic and farming systems and are major lentil-producing areas in the country. The lentil crop is important in these study sites and the Ethiopian context, given their significant role in income generation and providing better nutrition for HHs; improving soil fertility and its straw is key animal feed. Lentil varieties within the region mainly comprise of the local variety (Habesha), and new/improved varieties that have been introduced over the last two decades by Debre Zeit Agricultural Research Center. The major new/improved varieties include Alemaya (FLIP-89-63L), Derash (Alemaya/FLIP88-41L//02-AK-14) and Teshale (FLIP-96-46L). Lentil farming in both regions is rain-fed, and seeds, labour, fertilizers, herbicides, and pesticides are the main inputs used [25].

## Methods

A mixed methods approach comprising surveys, in-depth interviews and focus group discussions with multistakeholders (farmers, traders, agro-processors, and lentil breeders) was used. The study employs an intersectional lens that focuses on gendered, socioeconomic and household (HH) determiners of lentil trait preferences and adoption, given the existing literature gap in this area. Mixed methods were chosen to enable a broader and deeper examination of lentil trait preferences and adoption [39]. Data for the study were collected between July 2018 and December 2020. Farmer participants/groups identified for the study were farm managers and/or persons who are mainly responsible for attending to crops on the farm.

For the quantitative strand, a structured, closed-ended survey was administered to 280 farmers (140 women and 140 men) in Amhara and Oromia regions. Survey questions asked participants about lentil varieties cultivated, preferred traits in these varieties, and the underlying reasons for these preferences. Survey participants were also asked questions about lentil marketing and sales. Regarding the qualitative component, 214 in-depth interviews (IDIs) and 24 focus group discussions (FGDs) were held in the two regions. A total of 528 informants (250 women and 278 men) were recruited for our study. FGDs had an average of 10 members per group, and participants for the qualitative component included farmers, agro-processors, traders, and lentil breeders (Table 1). Qualitative interviews were semi-structured and facilitated with interview guides. Questions were open-ended and asked participants about their lentil trait preferences, reasons for growing or not growing lentil varieties, and experiences of engaging with other stakeholders (e.g., agro-processors, and traders) in the lentil value chain. For participants who were not farmers (e.g., lentil breeders, agro-processors), IDI questions asked about their engagement with farmer groups and their priorities/preferences in lentil varieties. Anonymity and informed consent were followed in accordance with ICARDA research regulations. All participants were aged 18 or older, and verbal consent was obtained from each participant before data collection began. Once consent was obtained, a check was marked next to a statement entitled “informed consent obtained” to document the participant’s agreement, and the interview commenced only afterward. This process was approved by ICARDA’s Institutional Review Board (IRB), with oral consent being documented through written form and witnessed by the enumerator and the respondent.

**Table 1:** Number of participants in study.

		Number of Participants					
Research Instrument Type		Group	Women		Men		Total
**Qualitative Data**	**IDIs**	Lentil Farmer		87		83	170
		Sharecropper		5		10	15
	***IDI Sub-total***			**92**		**93**	**185**
	**Key Informants**	State/non-state institutions		2		11	13
		Agro-processors		–		2	2
		Facilitators		1		3	4
		Breeders/Programme Leaders		–		2	2
	***Key Informants Sub-total***			**3**		**18**	**21**
	**Traders**			1		7	8
	**Total**			**250**		**278**	**528**
**Quantitative Data**	**Interviews for Quantitative assessments**	W-MHH		79			79
		WHH		61			61
		MHH				140	140
	**Total**			**140**		**140**	**280**

MHH = man-headed HH; WHHH = women-headed HH, W-MHH = women in male-headed HH.

Quantitative data were analyzed using descriptive statistics, with the help of the Statistical Package for the Social Sciences (SPSS), and results were presented using percentages and means/averages. Qualitative data were analyzed using open coding and with NVivo, the QSR software for qualitative analysis. Theme identification was the dominant analytical technique employed in coding. Specifically, using inductive content analysis [40], the study generated themes after coding and categorizing concepts based on their dimensions [41], using NVivo 10. Results from the qualitative data are presented using themes and direct quotations. The quantitative and qualitative data and results are integrated using explanation building, to understand and report the data. Based on quantitative data, we were interested in examining the trait preference of lentils in Ethiopia by asking the following question: What are the preferred characteristics you look for in a lentil crop (list the top 3)? The answers received were sorted and organized in groups, as a result, nine characteristics were evoked. These are (1) market value; (2) disease resistance; (3) good productivity; (4) compatibility with the soil; (5) fast growing; (6) good taste; (7) easy farming work; (8) good residue, and (9) cold resistance.

In order to study concerns about gender-related traits, we studied each trait separately. We assigned 1 for a positive response given to a trait, otherwise, we assigned 0 if not. And then we run chi-square and Fisher’s exact tests on the trait responses to identify significant differences between genders. When the sample size is too small it is more accurate to use Fisher’s exact test than the chi-square test. Statistical Package for Social Sciences (SPSS) version 26 was used to compute the test statistics. For the continuous variable on the characteristics of the group of farmers around the production and sale of lentils, we used the ANOVA test to detect significant differences when treating marital status groups (3 groups) and T-test when treating gender groups (2 groups). The distinction between tests is because one-way-ANOVA is used when we have 3 or more groups and T-test is used when we have two groups.

## Findings and discussion

**Fig 1:**
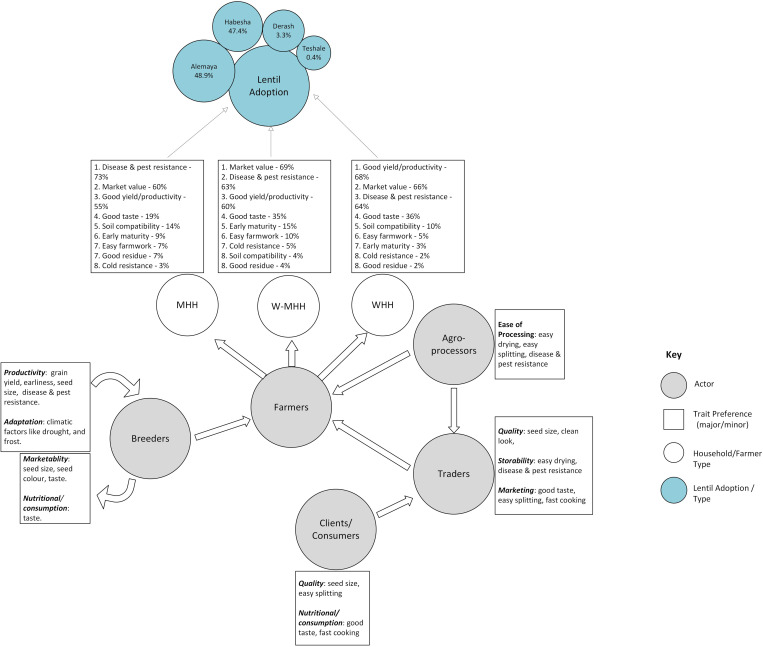
Lentil adoption preferences and stakeholder network (Source: Authors illustration).

Reflective of our theoretical perspective, most findings were segregated into three farmer groups by gender and HH type. The three groups are men-headed HHs (MHH), women-headed HHs (WHH), and women in men-headed HHs (W-MHH). W-MHH and WHH respondents are all women, whereas MHH respondents are all men. The results from the quantitative and qualitative data are presented interchangeably, and results from the respective strands are used to elaborate upon findings. The section begins with the findings on characteristics of the various lentil varieties, as well as farmers, traders and agro-processors lentil trait preferences. It then goes on to present the results on lentil adoption among farmers, socioeconomic considerations in lentil farming, and the perspectives of breeders regarding lentil trait preferences and adoption. See Fig 1 “Lentil Adoption Preferences and Stakeholder Network” that illustrates the interconnected network of actors involved in lentil adoption, highlighting the preferences of farmers, processors, traders, and consumers regarding key traits such as productivity, marketability, adaptation, and nutritional qualities.

### Lentil traits and preferences among farmers

#### Lentil varietal traits.

During the IDIs, farmers were asked to indicate the major lentil varieties grown in the two regions and provide the different features of these varieties including seed size, seed coat and cotyledon colour, selling price, and positive and negative characteristics. Findings from the qualitative data are presented below (Table 2). The qualitative results revealed that only 7% (*n* = 93) of men and 14% (*n* = 92) of women grew Derash, while no farmers grew Teshale – both new varieties. The data also showed that traits relating to productivity (yield, disease resistance, fast growth/early maturity), marketability (good market demand, good selling price), quality in processing (large seeds), quality in consumption (good taste and flavor, fast cooking time), and good fodder for livestock, were mentioned as the positive characteristics in lentil varieties. The absence of these traits, on the other hand, was considered a negative trait in lentil varieties. More women than men grew Derash due to it liking less inputs.

**Table 2:** Lentil trait characteristics among farmers.

Characteristic						
Group	Lentil variety	Seed coat colour	Price per Kg^ (whole seed) ETB	Price per Kg^ (split seed) ETB	Positive characteristics	Negative characteristics
**Farmers** **(W**-**MHH, WHH and MHH)**	Alemaya	Light	30–50	55–70	- Good yield - Good straw/fodder - Fast growth/Early maturity - Large seed - Fast cooking time^†^	- Susceptible to disease - Poor market demand * - Low market price† - Poor taste *
	Local/Habesha	Dark	49–85	75–95	- Good taste and flavour - Familiarity - Good selling price - Good market demand - Fast cooking time	- Poor yield - Susceptible to disease - Susceptible to frost - Poor straw - Small seed
	Derash	Gray	35–40	50–65	- Diseases resistant * - Fast growth/Early maturity† - Low inputs (fertilizer & labour)† - Cost effective†	- Small seed - Poor market demand * - Poor yield† - Poor splitting†
	Teshale	Red	N/A	N/A	N/A	N/A

* Mentioned only by women.

^ Kg denotes kilogram.

† Mentioned only by men.

Evidence from recent studies show that despite modern lentil varieties having significantly higher yields, they did not lead to better adoption rates, compared to the local lentil variety. For instance, exotic lentil germplasm from ICARDA and ACIAR have shown significant yield enhancements, with yields up to 66.7 grams per plot compared to 22.8 grams for local varieties [41,42]. However, their longer maturity times make them vulnerable to environmental challenges, emphasizing the need for breeding programs that integrate early maturity traits from local landraces.

In this study, despite the unanimity across farmer groups about positive and negative lentil attributes, these attributes were sometimes associated with different lentil varieties by the different groups. For instance, although fast cooking was identified by both women and men farmers as a positive trait, only MHH associated this trait with the Alemaya variety. Interestingly, only women (W-MHH and WHH) identified poor market demand as a negative trait and associated this quality with the Alemaya and Derash varieties, whereas only men (MHH) identified low market price as a negative characteristic. Further, while both women and men farmers identified good taste as a positive characteristic, only women identified poor taste as a negative trait and associated this with the Alemaya variety. Importantly, both women and men in all HH types were unanimous about the traits that were considered positive and negative in the local variety of lentils.

#### Farmer trait preferences.

When asked about the traits that they most preferred in the lentil crop, four traits were mentioned across all farmer groups, although the proportions of farmers who picked these traits differed by group. The four traits were market value, disease resistance, good productivity, and good taste. A few MHH (14%) and WHH (10%) identified soil compatibility as a preferred trait, and a few W-MHH (15%) identified fast-growing as a preferred trait (Table 3). Specifically, the traits most preferred by MHH are disease resistance (73% of all men), market value (60%) and good productivity (55%). The traits most preferred by WHH are good productivity (68%), good market value (66%) and disease resistance (64%). Finally, W-MHH prefers market value first (69%), disease resistance second (63%) and good productivity third (60%). A noteworthy difference is that more women prefer good taste (35% and 36% for W-MHH and WHH, respectively), compared to MHH where only 19% of participants selected this trait.

**Table 3:** Preferred characteristic in lentil crop by farmer group.

Group.	Preferred Lentil Traits										
		Market Value	Disease Resistant	Good Yield/ Productivity	Soil Compatibility	Early Maturity	Preferred Taste	Easy Farm Work	Good Residue	Cold Resistant	Total N = 280
W-MHH	N	54	49	47	3	12	27	8	3	4	79
	%	69%	63%	60%	4%	15%	35%	10%	4%	5%	
WHH	N	39	38	40	6	2	21	3	1	1	61
	%	66%	64%	68%	10%	3%	36%	5%	2%	2%	
Total Women	N	93	87	87	9	14	48	11	4	5	140
	%	66%	62%	62%	6%	10%	34%	8%	3%	4%	
MHH	N	84	102	77	19	12	27	10	10	4	140
	%	60%	73%	55%	14%	9%	19%	7%	7%	3%	
Total	N	177	189	164	28	26	75	21	14	9	277
	%	64%	68%	59%	10%	9%	27%	8%	5%	3%	
Pearson Chi-Square		0.460	0.166	0.384	0.071*	0.054*	0.019**	0.505	0.226	0.510	
Fisher’s Exact Test		0.460	0.154	0.384	0.053*	0.056*	0.017**	0.501	0.255	0.608	

The Fisher’s exact test is used instead of the Chi-Square Test when the cells have less than 5 observations.

***,**,*  denotes significance at the 1%, 5% and 10% level.

These findings emphasize the centrality of lentils as both a subsistence and market crop, and the resulting need to boost lentils production across all farmer groups, as evidenced by the importance of disease resistance, market value and good productivity as positive traits for all farmers. However, the difference in proportions among women and men farmers regarding good taste as a preferred trait in lentil varieties may likely be due to the gendered roles and responsibilities of farmers [43]. Specifically, the need for women farmers to meet HH food needs, and hence the similarity among both W-MHH and WHH for good taste as a preferred trait in lentils. Moreover, the fact that both MHH and WHH identified soil compatibility is indicative of adaptation in the farming system as a preferred trait, whereas W-MHH picking fast-growing may speak to the gendered differentiation of farm needs among diverse HHs. Thus, W-MHH may be less concerned about soil (to meet farming responsibilities), and more concerned about fast-growing (to meet HH food needs), whereas WHH is concerned about both, as they must meet both breadwinner and caregiving responsibilities. These findings support those of other studies such as [14,16,19] who found in their respective studies that women are often responsible for care provision, including food preparation, and may therefore take more interest in quality (e.g., taste) and early maturation of food crops, compared to men. The findings however differ from those of the aforementioned studies, as they emphasize within-gender differences, mediated by HH type.

As shown in Table 3, the soil compatibility trait was significantly different between marital status groups at the 10% level for both tests (Fisher’s exact test and chi-square test). When referring to the distribution of responses, we note that MHH expressed more interest in this trait. Early maturity trait difference analysis gave also the same level of significance but this time to suggest that W-MHH were more interested in this trait. The trait ‘good taste’ was more important for women in both groups (W-MHH and WHH) than for MHH (the difference was significant at a level of 5% in both tests). The same tests were performed according to gender instead of marital status groups enabling us to identify one more significant difference at the 10% level: disease resistance trait, with men more concerned by this trait (72.9% (N = 140) vs 62.1 (N = 140)).

#### Traders and Agro-processors’ lentil attributes and preferences.

Other stakeholders, specifically traders and agro-processors, were also asked to indicate the major lentil varieties grown and provide the different features of these varieties (Table 4). Again, the findings reveal cross-cutting positive and negative traits relating to quality (seed size), marketability (price, demand), processing (ability to split), and productivity (yield, resistance to disease/pest) among the various stakeholders. The IDIs with traders and agro-processors reveal that many farmers are disinterested in growing the Teshale variety, thereby supporting the findings from interviews with farmer groups.

**Table 4:** Lentil trait characteristics by other stakeholder groups.

Characteristic						
Group	Lentil variety	Seed coat colour	Price per Kg^ (whole seed) ETB	Price per Kg^ (split seed) ETB	Positive characteristics	Negative characteristics
**Agro-processors**	Alemaya	Light	36	58	- Large seeds - Split easily - Good straw	- Lower market price - Low demand - Less market value compared to local variety
	Local Variety	Dark	42	72	- Good market demand - High selling price.	- High milling losses - Small yield - Loss during splitting
	Derash	Gray	40	65	N/A	- High losses/small yield - Lower market price - Small seeds. not processed
	Teshale	Reddish	35	40	N/A	- Low market price - Small yield
**Traders**	Alemaya	Light	36–42	58–60	- Good yield - Large seeds - Splits easily - Good straw - Attractive seeds	- Poor taste - Not good for stew - Lack of consumer interest
	Local Variety	Dark	42–55	65–75	- Good taste - Preferred by consumers - Good for stew	- Low yield - Small seed - Poor splitting - Expensive price
	Derash	Gray	35	40–50	- Disease resistance - Fast growth/Early maturity - High yield	- Lack of consumer interest - Small seed - Unattractive seed
	Teshale	Reddish	35	45	- Large seed - Attractive seed	- Lack of farmer interest

^ Kg denotes kilogram.

The study also sought to understand the preferred traits in different lentil varieties among the above-mentioned stakeholders. The results show that traders prefer quality, storability, and marketing (consumer) traits such as good seed size, clean look, easy drying, easy splitting, resistance to disease/pests, good taste and fast cooking. Traders indicated that these trait preferences mainly result from consumer and agro-processor demands. For instance, when asked about preferred traits, a trader speaks to the ways in which customer preferences influence their own preference:

*For us traders, the quality of the crop depends on its cleanliness, absence of soil or rocks and not shrivelled by cold and rust diseases. People ask for fast-cooking lentils often, so how fast it cooks is also important. Another trait is taste. Customers say the dark lentil is tasty. Although some report that dark lentils can increase acidity in the stomach, it is still high in demand (Trader, Woman, 47, East Shoa Zone).*

For agro-processors, traits that make the processing of lentils easy, such as the ability to dry and split easily, and free from disease/pests are the most preferred. According to agro-processors, Alemaya and Teshale are the lentil varieties that split easily. Of the two, however, processors prefer Alemaya, given Teshale’s low market demand. The low market demand for Teshale may also be influencing farmers’ reluctance to grow it, given that marketing traits are considered important for lentil farmers. These results therefore highlight the interconnectedness of trait preference across different stakeholder groups and support the findings of Forsythe et al. (2021) who argue for the importance of considering the diversity and effects of other food stakeholders’ product preferences on the success of crop varieties.

#### Lentil varietal adoption among farmers.

An objective of this study was to examine rates of adoption among farmers, to understand how negative and positive traits of lentil varieties, as well as the preferences identified by farmer groups (as discussed above), may be influencing their adoption rates. To achieve this. farmers were asked about the lentil varieties that they grew in the long season (lentils are only grown during the long season). The findings are presented in Table 5 below.

**Table 5:** Lentil adoption rates among farmer managers.

Lentil variety grown in long rain season						
Group		Alemaya	Local Variety	Derash	Teshale	Total (N)
W-MHH	N	37	37	1		75
	%	49.3%	49.3%	1.3%		
WHH	N	31	29	1		61
	%	50.8%	47.5%	1.6%		
MHH	N	65	63	7	1	136
	%	47.8%	46.3%	5.1%	0.7%	
Total	N	133	129	9	1	272
	%	48.9%	47.4%	3.3%	0.4%	

The results showed that 48.9% of all farmers grow Alemaya, one of the improved varieties. However, this is only 1.5% more than the proportion of all farmers who grow the local variety (47.4%). A study conducted in 2019 indicated that the total land area dedicated to local lentil cultivation in Ethiopia has increased at a compound growth rate of 4% per annum, with production volumes rising by 9% per annum over the study period [44]. Likewise, the productivity of lentils has shown a compounded growth rate of 5% per annum. However, in Ethiopia’s central highlands, only 9% of lentil farmers have adopted improved varieties, largely because of challenges related to diseases and insect infestations, compounded by gender inequalities [45]. This is against the backdrop that improved lentil varieties have demonstrated yield advantages of two to three times more than local varieties [46]. Women farmers face greater challenges in adopting improved lentil varieties due to limited access to resources such as quality seeds, fertilizers, pesticides, credit, and information, underscoring the need to pay attention to the gendered differences in adoption. To put this gender difference in perspective, in Oromiya, 50% of female-headed households experienced lentil losses, compared to 28.6% of male-headed households [47].

Consistent with the literature, our data show that only three and less than one percent of farmers grow Derash and Teshale (the other new lentil varieties), respectively, and the majority of these are MHH. Irrespective of the similarities in adoption rates across all farmer groups, WHH and MHH both report growing slightly more Alemaya than the local variety, whereas W-MHH report growing equal quantities of Alemaya and the local variety. The data thus suggests that there are no significant sex or marital status-related differences in the adoption of these improved lentil varieties. Rather, these findings highlight the fact that many farmers in the two regions are still growing the local lentil variety, emphasizing the relatively low rates of adoption of the improved varieties, such as Alemaya, Derash and Teshale—which are considered ‘improved/modern’ varieties. This finding supports those of previous studies that found that several farmers choose to still grow local crop varieties (sometimes alongside newer varieties), even with the introduction of modern varieties [1,3,6,7].

Qualitative interviews with farmers’ groups sought to unearth the underlying reasons for these low adoption rates. The findings reveal that many farmers are still growing the local variety because it possesses many of the traits that they prefer in the lentil crop. These traits include good taste and flavour, good market demand, good selling price, and fast cooking time. Many farmers also indicate that their familiarity with producing the local variety is one of the reasons why they still produce it in large quantities. Farmers add that for them, the positive characteristics of the local variety override its negatives such as poor yield, susceptibility to diseases and frost, and lower residue production. They further indicate that their decisions are rooted in the fact that some of the newer/improved varieties come with the same negative characteristics (e.g., susceptibility to diseases) as the local variety, and additionally, have poor taste, low market demand, and more labour and input requirements. For instance, a woman farmer says:

*I grow only the local lentil (Habesha). I stopped growing Alemaya a few years back. The local lentil requires less labour and effort to grow and pick. The Alemaya variety was good previously. now it is losing its benefits (Woman, 50, farmers group member, North Shoa-Zone).*

These findings on the low adoption rates of the new varieties perhaps explain the consensus about the negative and positive traits of the local lentil among all farmer groups, which was not the same for the newer lentil varieties. The findings are also consistent with studies that note that farmers may show risk averseness to modern crop varieties – particularly if these newer varieties come with low profitability – motivating them to keep producing varieties that they are familiar with [3,6,10].

#### Intersectional considerations in lentil production.

Finally, this study also aimed to understand how socioeconomic characteristics (e.g., farm size and access to inputs), HH type (e.g., size, male-headed, or female headed), and broader agricultural conditions (e.g., policies. market access) may be influencing lentil production and sale across diverse farmer groups. To do so, several production and marketing characteristics of the various farmer groups were collected. These characteristics are presented in Table 6 below, and a few important results are discussed briefly.

**Table 6:** Farmer group characteristics around lentil production and sale.

Group	Characteristics									
		Age (years)	Phosphorous (DAP)	Area of land owned (ha)	Area size of lentil (ha)	Yield quintals/ha cultivated	Amount of crop residue quintals/ha cultivated	Revenue (Birr/ quantal)	Grain sold (%)	Consumed by HH* (%)
W-MHH	Mean	41	168	1.5	0.5	14.5	32.8	6309	75.6	4.0
	Min	23	0	0	0.25	0	0	0	0	0
	Max	60	2800	6	1.5	64	180	40000	100	60
	N	40	44	79	66	66	46	64	68	68
WHH	Mean	42	149	1.3	0.4	16	31	5159	70.3	4.7
	Min	28	0	0.3	0.3	0	0	0	0	0
	Max	65	800	4	1.5	200	120	55000	100	100
	N	30	45	61	60	60	45	55	56	56
Total Women	Mean	43	158	1	0	15	14	5778	73	4
	Min	20	0	0	0,25	0	0	0	0	0
	Max	65	2800	6	1,5	200	90	55000	100	100
	N	140	89	140	126	126	93	119	124	124
MHH	Mean	44	177	1.3	0.6	17.3	48	10013	80.8	6.1
	Min	20	0	0	0.3	0	2	0	0	0
	Max	78	1600	6	6.5	80	360	90000	100	100
	N	68	110	140	134	134	101	129	132	124
Totals	Mean	43	169	1.4	0.5	16.3	40.4	7981	77.1	5.2
	Min	20	0	0	0.25	0	0	0	0	0
	Max	78	2800	6	6.5	200	360	90000	100	100
	N	138	199	280	260	260	192	248	256	248
Significance of difference using One-way ANOVA test 1		0.133	0.841	0.157	0.168	0.545	0.008***	0.022**	0.108	0.528
Significance of difference using T-test 2		0.99	0.629	0.331	0.070*	0.326	0.002***	0.007***	0.057*	0.274
	1 **USD = 49.05 Ethiopian Birr**									

* Household.

^1^Used when comparing between marital status groups (three groups).

2Used when comparing between gender groups (two groups).

***, **, *  denotes significance at the 1%, 5% and 10% level.

From the results, WHHs on average use the least quantity of inputs such as phosphorus/urea (149), compared to MHH (177) and W-MHH (168) for their lentil crop. Counterintuitively, W-MHH owns more land on average (1.5), while MHH and WHH own the same land sizes on average (1.3). Also, WHH allocates the least land area on average (0.4) to lentil cultivation. As compared to W-MHH (0.5) and MHH (0.6). Interestingly, however, W-MHH and WHH are producing less lentil seed (14.5 and 16 respectively) and straw (32 and 31 respectively) per area size compared to MHH 17.3 for seed and 48 for straw. This is despite owning more and the same hectares of land on average as MHH. This may be attributed to the fertility of the lands owned by the respective groups or the influence of input use on lentil productivity. This finding of lower yield supports those of earlier studies which show that men may be more advantaged in terms of being able to access and use chemical and complementary inputs (e.g., fertilizers, fertile land, and credit) thereby enabling them to be more productive in farming and technology adoption [2,8,14].

These disparities are even more pronounced in terms of the revenue accrued from the sale of lentils, with MHH earning about twice (10.013 Birr) what W-MHH and WHH earn (6.309 and 5.159 Birr, respectively), even though the gaps in production are not as wide. The fact that women are earning less for lentils may explain why they are the only ones to identify poor market demand as a negative characteristic of lentil varieties. The study results also reveal that MHH sells larger proportions of their lentil produce on average (80.8%), compared to W-MHH (75.6%) and WHH (70.3%). This may be evidence that MHH is engaging in more market-oriented farming. as compared to women farmer groups (W-MHH and WHH). Lastly, the findings show that W-MHH and WHH consume less lentil on average, which is likely explained by the fact that they make lower yields. At the national level, smallholder farmers use their lentils for home consumption (41%), sales (39%), as seed (18%) and the rest for other needs [48]. These findings support those of earlier studies such as [49–51] who demonstrate that men tend to benefit more not only in production but in the marketing and sale of crops, including commercializing ones.

The one-way ANOVA test in Table 6 further revealed that the difference was significant between marital status groups at the 1% level for the amount of crop residues cultivated and at 5% for the income of the total amount sold. For these two variables, MHH had higher values (means were higher) compared with both groups of women (W-MHH and WHH). The same two characteristics, i.e., crop residue and income, were also significantly different at 1% when comparing between genders. Using the T-test, as expressed in Table 6, in place of two other variables: the area of lentils and the total percentage of grain sold which were significantly different at 10%, men had higher means compared to women.

#### Trait preferences and breeding priorities.

Another important goal of this study was to understand whether farmer and other stakeholder preferences inform the priority of varietal breeding programs. Interviews with breeders in Ethiopia suggest that, although some farmer engagement and feedback are integrated into varietal breeding, this engagement is limited. There is still a disconnect between the traits that farmers value in a crop versus the traits that are considered the most important by breeders. Generally, breeders mentioned that new lentil varieties are required to exceed previous varieties by at least 10% in terms of quantity/agronomic traits. When asked about the specific traits that they breed for, one breeder mentioned mainly productivity traits such as grain yield, earliness, seed size and disease resistance. The second breeder also mentioned productivity traits such as disease and pest resistance, and adaptation to climatic factors like drought, and frost. However, the second breeder included market traits (such as seed size, seed colour, and taste) and nutritional and consumption traits (including taste) as qualities they breed for. Both breeders shared a consensus that productivity and market traits were nonetheless the most important ones for them. However, nutritional and consumption quality are often included as add-ons or relegated altogether.

Discussions with breeders further reveal no consultation with other important stakeholders (e.g., agro-processors, traders) within the lentil value chain in the development of new crop varieties. Breeders admit that even the few farmer engagements tend to lack a gender balance, with male farmers being the most consulted group. These admissions are consistent with the findings of recent studies highlighting that seed breeding and multiplication initiatives tend to under-recognize and underserve women farmers [52–54]. As reasons for the low engagement with women, farmers breeders cited (1) men’s ‘dominance’ in farm work, (2) female farmers’ reluctance to participate in varietal selection field activities, and (3) women’s multiple farm and caregiving responsibilities that leave them with little to no time to participate in consultation meetings. One breeder admitted that in the few instances that both women and men were consulted on varietal selection, women tended to pick more consumption quality traits, while men often leaned towards agronomic traits such as productivity. However, the traits chosen by men farmers are the ones ultimately prioritized in breeding new varieties. Consequently, breeders appear to be focusing more on improved varieties such as Derash and Teshale. These varieties possess the traits that they (breeders) consider important (large seed size, high yield, and disease resistance). However, these varieties lack traits – good taste, splitting, market demand and price – that most stakeholders, including farmers, agro-processors and traders prefer.

## Conclusion

This study examined gendered trait preferences and adoption among lentil farmers in Ethiopia. It also explored how the preferences of other stakeholders such as traders and agro-processors influence farmers’ preferences and adoption of different lentil varieties. The findings reveal similar yet distinct preferences across farmers of different genders and HH types. The findings further show that most farmers still produce the local lentil variety, despite the introduction of modern/improved varieties. Farmers cite factors such as poor taste, poor disease resistance, poor market demand and low prices of modern varieties as reasons for not growing them. In addition, farmers prefer lentil varieties that have good taste, and easy processing and storability as they are the traits valued by customers, agro-processors, and traders. The study findings also reveal that, on average, W-MHH and WHH may not have the same access to complementary and chemical inputs such as land and fertilizers as MHH. This limited access leads to lower yields, consumption and income accrued from lentils for women groups.

Thus, while breeding and adoption programs are aimed at improving the quality of staple foods, and food and economic security, these programs may be falling short of meeting these goals due to the inequitable benefits realized from breeding and adoption programs, mainly resulting from a lack of gender balance in breeding goals. The lack of consultation with other important stakeholders also leads to a disconnect between farmer and stakeholder needs and the priorities of breeding programs.

To address these gaps, lentil breeding programs need to go beyond agronomic traits such as good yield/productivity. Breeding programs need to include other important traits such as consumption quality (e.g., good taste). These traits are preferred by farmers, traders and customers. It is interesting that all improved varieties were introduced and selected from ICARDA lentil breeding program without crossing with local preferred varieties. However, it is advisable to include local landraces in the cropping program to capture traits revealed by farmers in this study. It is also important for breeding programs to consider how resource constraints such as poor input access and use may influence resource-poor farmers’ ability to adopt newer varieties that require more input use to boost productivity. Moreover, it is crucial for breeding programs to undertake equitable and gender-responsive breeding. This is to ensure that the trait preferences of diversely situated groups are taken into consideration in the development of new varieties. It is also necessary for breeders to understand how the perspectives and preferences of all stakeholders within the lentil value chain influence farmers’ own preferences and adoption. This is to ensure that modern crop varieties better suit the needs of all. It is advisable to include straw yield and palatability to livestock since varietal adoption can be affected by this trait given its shortage in mixed farming systems of Ethiopian highlands [55]. Finally, it is important to create more equitable markets for women and other resource-poor farmers in Oromia and Amhara, and Ethiopia more broadly. Markets enable them to better benefit from growing market-oriented lentil farming in the country.
